# Dr. Jason T. Wallace

**Published:** 1918-01

**Authors:** 


### OBITUARY

**Readers are requested to report promptly the death of all physicians in Western New York, or former residents of this region, or graduates of any medical school in Western New York, and to notify the families of the deceased of our desire to publish adequate Obituary notices.**

**Dr. Jason T. Wallace**, Oneida, N. Y., New York Homoeopathic Medical College, New York City, 1865, age 81, a member of the first board of education and of the board of health of Oneida, died in that city November 16 from heart disease.

